# Physical properties and cytotoxicity of silver nanoparticles under different polymeric stabilizers

**DOI:** 10.1016/j.heliyon.2019.e01305

**Published:** 2019-03-07

**Authors:** Roman Verkhovskii, Anastasiia Kozlova, Vsevolod Atkin, Roman Kamyshinsky, Tatyana Shulgina, Olga Nechaeva

**Affiliations:** a School of Urbanistics, Civil Engineering and Architecture Chair of Ecology, Yuri Gagarin State Technical University of Saratov, Russia; b Department of Nano- and Biomedical Technologies, Saratov State University, Russia; c National Research Center “Kurchatov Institute”, Akademika Kurchatova pl., 1, 123182, Moscow, Russia; d Shubnikov Institute of Crystallography of Federal Scientific Research Centre “Crystallography and Photonics” of Russian Academy of Sciences, Leninskiy prospect, 59, 119333, Moscow, Russia; e Institute of Traumatology and Orthopedics, Saratov Medical State University, 410002 Russia

**Keywords:** Nanotechnology, Materials science

## Abstract

At present day, silver nanoparticles are widely used in different fields of human activity. Due to the unique combination of physical and chemical properties, silver nanoparticles have high reactivity and antibacterial activity against microorganisms. For the same reason, silver nanoparticles can render a cytotoxic effect on eukaryotic cells. The usage of different polymeric compounds as stabilizers can allow reducing of it and saving antibacterial activity. With this regard, the examination of new nanoparticles' stabilizers is a vital task. In addition, for the safe usage of silver nanoparticles it is necessary to estimate some of their physical properties and cytotoxicity. Here we evaluated the shape, size, UV-visible absorption, fluorescence, z-potential and cytotoxicity of single silver nanoparticles and nanoparticles, stabilized by polyvinyl alcohol, sodium carboxymethylcellulose, sodium dodecyl sulfate, sodium oleate and agarose. We found that nanoparticles stabilized by all investigated polymeric compounds with the exception of sodium dodecyl sulfate and sodium oleate did not possess significant cytotoxic effect on the test cell culture.

## 1 Introduction

At present day, silver nanoparticles (Ag NPs) have found a wide application in many fields of human activity due to their unique physical and chemical properties, such as a small size, high specific surface area (the ratio of free surface area to mass), high reactivity etc [1, 2, 3, 4]. The ultrasmall size of Ag NPs and, as a consequence, a high specific surface area are one of the main reasons for their increased antibacterial and antiviral activity [5]. Ag NPs, like all nanostructures, have a size varying from 1 to 100 nm [2, 6]. Size reduction leads to an increase of their antibacterial activity [7]. Moreover, the effectiveness of Ag NPs application largely depends on the shape and nanoparticle concentration [8, 9, 10, 11]. Shape and size of Ag NPs can be changed by synthesis conditions variation [12]. That makes the development of new approaches to the Ag NPs production to be a very promising direction. One more reason of high interest to Ag NPs is the growing amount of pathogenic antibiotic-resistant strains of microorganisms [3, 13, 14].

Ag NPs are widely used in cosmetology [15, 16, 17], textile industry [18, 19], production of medical and household products [3, 20, 21] for food storage [22, 23] and some fields of science.

Due to their high reactivity, Ag NPs possess biocide effect against broad range of bacteria, such as *Escherichia coli*, *Staphylococcus aureus*, *Staphylococcus epidermis*, *Leuconostoc mesenteroides*, *Bacillus subtilis*, *Klebsiella mobilis*, *Klebsiella pneumonia*, fungi: *Aspergillus niger*, *Candida albicans*, *Saccharomyces cerevisia*, *Trichophyton mentagrophytes*, *Penicillium citrinum*, and some viruses: Hepatitis B, HIV-1, syncytial virus [24, 25]. Biocide properties provide to use Ag NPs for solutions of various purposes. For example, the gel-like composite implant, used for filling the tubular bone defect zone, contains a solution of zero-valent metallic Ag NPs [26]. In surgery, such nanoparticles and films with polyethylene glycol, glycerin, containing them, are applied as well to cover damaged skin areas [27, 28]. Nanocomposites materials with silver are commonly used as antimicrobial films for food packaging and its storage time increment [22]. In addition, Ag NPs are successfully used to improve the Surface-enhanced Raman spectroscopy (SERS) signal [29].

Although antibacterial properties of silver have been known for a long time [30, 31, 32] and Ag NPs have been successfully used as antibacterial agents, the mechanism of their influence has not been fully studied [5]. One of the reasons for their antibacterial activity is a release of Ag^+^ ions, which can interact with thiol groups of some bacterial proteins, playing important role in the DNA replication [33]. In addition, a release of silver ions can lead to uncoupling of two important cellular processes, such as electron transportation in the respiratory chain and oxidative phosphorylation of adenosine diphosphate (ADP) [34]. A number of publications reports that Ag NPs are able to damage the cell wall and cell membrane of bacteria, thereby leading to its death [14, 35, 36].

However, the properties, which allow us to use Ag NPs as an antibacterial agent, can also be active against eukaryotic cells. Even small doses of nanoparticles have the potential to cause DNA damage and chromosomal aberrations that leads to cell cycle arrest [37].

Nevertheless, the safety of their usage strongly depends on the state: many nanostructures may aggregate during their production and usage. It occurs due to the low surface charge that usually prevents particles from bunching. Also such nanostructures may aggregate during the formation process, if they covered by high viscosity substance or suspended in high viscosity environment [38].

Thereby, different chemical compounds, which can cover nanoparticles and used as stabilizers, have found a wide application in the field of nanoparticle production. One of the examples are polymeric compounds. We can control the surface properties of nanoparticles using stabilizers with different parameters, such as molecular weight, charge, chemical functionality and hydrophobicity. Here we described some physical properties of silver nanoparticles stabilized by various organic polymers, evaluated their cytotoxic effect depending on the used stabilizer and thus estimated the effectiveness and safety of particular silver nanoparticle stabilizers application.

## 2 Materials and methods

Silver nanoparticles, obtained by chemical reduction method (patent RU 2 638 716 C2), were provided by LLC "M9". Polyvinyl alcohol 0.7% (PVA), sodium carboxymethylcellulose 0.01% (CMC), sodium dodecyl sulfate 0.15% (SDS), 0.15% sodium oleate (Ole Na) and 2% agarose (AgA) provided by LLC "M9" were used as stabilizers. Minimum Essential Medium (MEM, Sigma-Aldrich), fetal bovine serum (FBS, Hyclone), penicillin-streptomycin antibiotic antifungal cocktail (Sigma-Aldrich), 0.05% trypsin with EDTA (Life technologies). Ultrapure water (resistivity >18.2 MΩ·cm) was used for all experiments.

### 2.1 Electron microscopy

Particle morphology characterization was investigated by the scanning (SEM) and transmission (TEM) electron microscopy. SEM measurements were performed via MIRA II LMU (Tescan) at the operating voltage of 30 kV, in second electron and back scattering electron modes. In the process of measurement, magnification was ranged from 100 to 40.000 times. Transmission (TEM) electron microscopy imaging was performed with a Titan 80-300 TEM/STEM (FEI, USA) electron microscope, equipped with a Schottky field emission gun, spherical aberration corrector (Cs probe corrector) and energy dispersive X-ray spectroscopy system (EDXS; EDAX, USA). Samples were prepared by drying a drop of the aqueous suspension of sample on the carbon coated Lacey copper grid.

### 2.2 Measurements of ζ - potential of silver nanoparticles

Zeta potential of Ag NPs and nanoparticles, stabilized by different polymeric compounds, were measured using a Zetasizer Nano ZS instrument (Malvern Instruments Ltd, UK) equipped with a 532 nm laser. The analysis was performed at 25 °C with use of corresponding refraction indexes. For zeta potential measurements, particles were suspended in ultrapure water or polymer solutions.

### 2.3 UV-Visible and fluorescence spectroscopy

UV(ultraviolet)-visible absorption and fluorescence spectra of native particles and treated by different stabilizers were carried out using a configurable multi-mode microplate reader Synergy H1 (BioTek, USA). Absorption spectra were carried out in the range of 300–900 nm with a step of 2 nm. Fluorescence spectra were carried out in the range of 380–700 nm at 350 nm excitation with a step of 2 nm.

### 2.4 Cell preparation

Сell culture of normal human dermal fibroblasts (NHDF) were provided by the Department of Cell Engineering, Education and Research Institute of Nanostructures and Biosystems, Saratov State University, Russia. Cell culture were plated in tissue culture flasks and cultivated in MEM, containing 10% of FBS, and 1% of penicillin-streptomycin antibiotic antifungal cocktail. The medium was replaced every 3 days. Cell culture were maintained in a humidified incubator at 37 °C with 5% CO_2_. After that, a monolayer of cell culture was harvested using 0.05% trypsin with EDTA and counted by the Countess™ automated cell counter (Thermo Fisher Scientific).

### 2.5 Cellular cytotoxicity

Estimation of cytotoxic effect was performed on the NHDF cell culture. Cells were seeded into 96-well culture plates at the amount of 10^4^ cells per well. After 24h cultivation, the test substance was added into the well with cells and incubated overnight at 37 °C under 5% CO_2_. After that, 10 μL of fluorescence dye (AlamarBlue, Sigma-Aldrich) was added to the each well with 100 μL of cultural medium. In the last step, fluorescent (560/590 nm) intensity was measured by a spectrophotometer (Synergy H1 Multi-Mode Reader).

## 3 Results and discussion

**Fig. 1:**
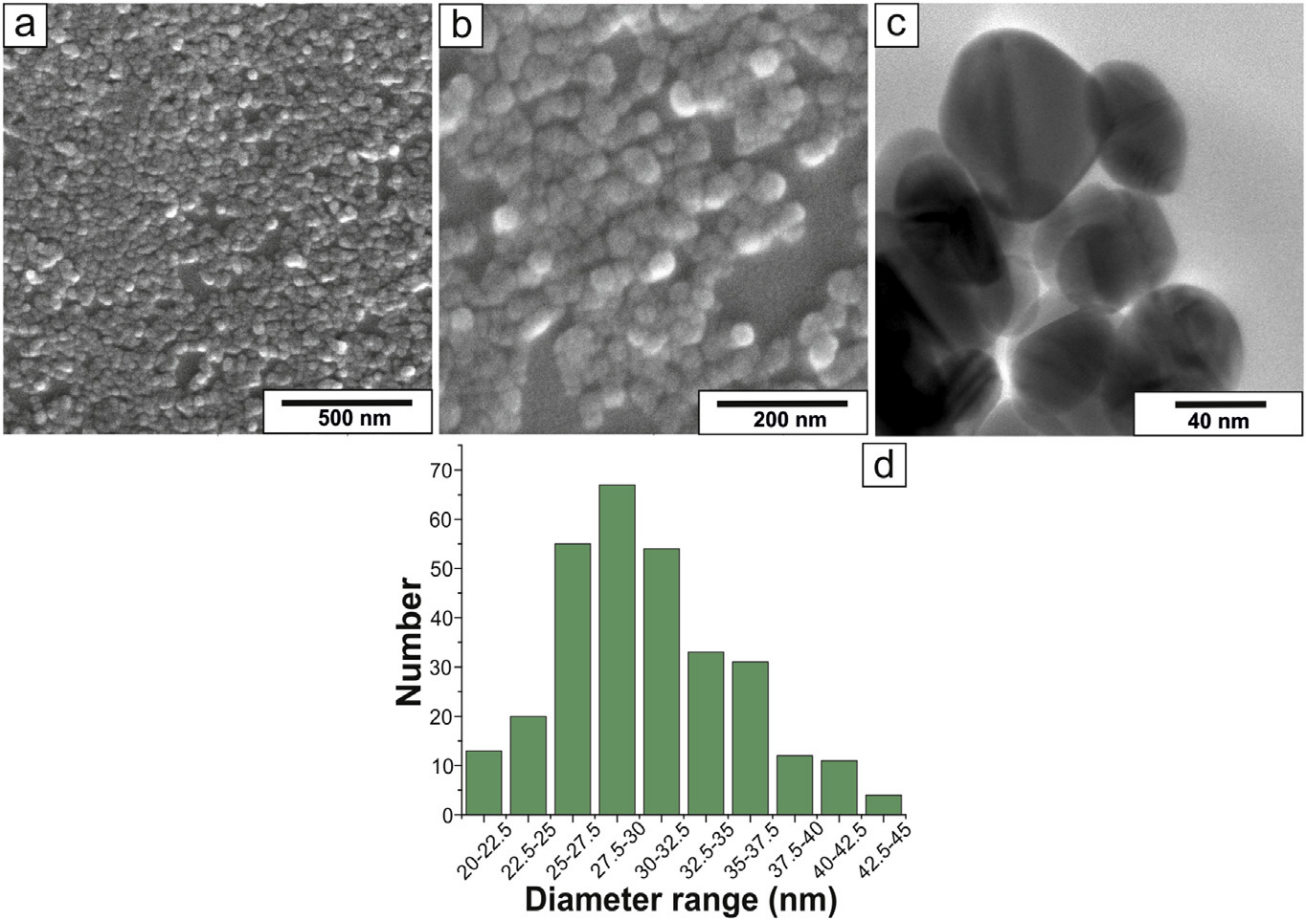
SEM (a, b), TEM (c) images of silver nanoparticles and their size distribution, obtained by SEM (d).

The biological activity of Ag NPs largely depends on the specific surface area of nanoparticles and consequently on their size [5, 37]. With this regard, we estimated the shape of Ag NPs by SEM and TEM (Fig. 1 a–c), and their size distribution by SEM images analysis (Fig. 1d). We found out that nanoparticles have a spherical shape and diameter, ranging from 20 to 45 nm. The major part of nanoparticles has a diameter from 25 to 32.5 nm and only a small part of them has extreme diameter values.

The spherical shape of Ag NPs allowed us to estimate their ζ - potential. These particles had a positive charge in water. Nevertheless, in stabilizers their potential was negative, according to the following results presented in the Table 1.

**Table 1:** ζ -potential of Ag nanoparticles dispersed in different solutions.

Dispersant	Refractive index	Zeta potential (mV)
CMC	1.515	−49.6 ± 3
SDS	1,461	−40.3 ± 6
Sodium Oleate	1,463	−70.7 ± 13
Agar	1,343	−21.3 ± 4
PVA	1,500	-5.8 ± 1.6
Water	1,333	17.2 ± 2

We found that Ag nanoparticles have the highest negative potential in sodium oleate. It effectively prevents them from aggregation and ensures the usage of sodium oleate to stabilize. Such stabilizers as CMC, SDS, and Agarose provide a significant negative potential of nanoparticles, which can prevent their bunching, while in PVA stabilized particles have the lowest ζ - potential. This may limit it's usage for nanoparticle stabilization.

It should be mentioned that silver nanoparticles strongly interact with light due to the conduction electrons on their surface, which leads to the surface plasmon resonance (SPR) and specific peak on the absorption spectrum. But, due to the Mie's theory describing the optical density dependence on particle's size [39], this peak can be easily tuned: with the increasing of particle size the peak of absorption becomes red-shifted and more faint [40, 41]. To estimate the effectiveness of silver nanoparticles stabilization, we investigated some optical properties of polymeric compounds and stabilized Ag NPs, such as UV-visible absorption and fluorescence.

**Fig. 2:**
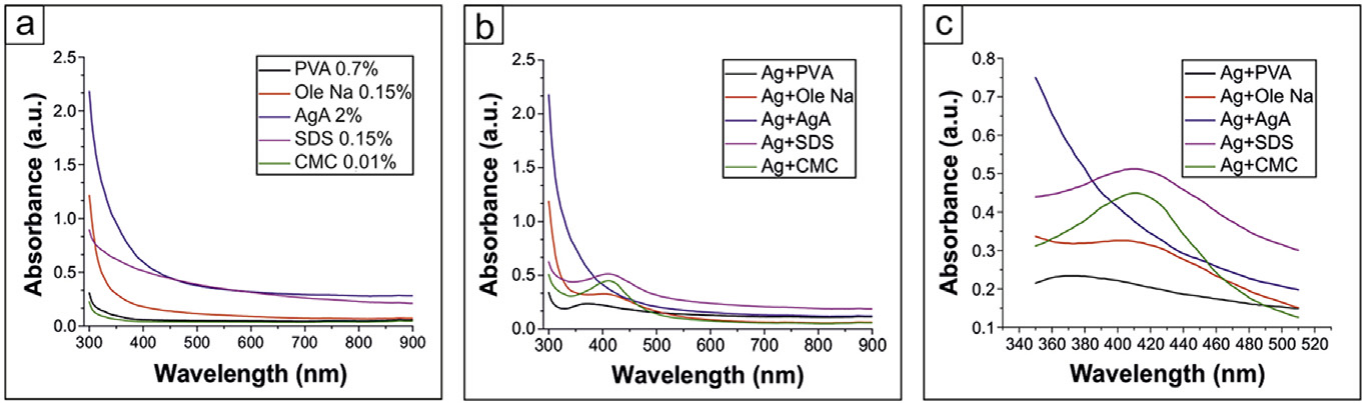
UV–visible absorption spectra of different polymer compounds (a) and Ag NPs, stabilized by them (b); the magnification spectra of stabilized Ag NPs absorption (c).

Absorption spectra measurements were carried out in the range of 300–900 nm. We established that the stabilizers do not have specific absorption peaks (Fig. 2 a). This means their spectra will not overlap the specific absorption peak of silver, which allow us to estimate the efficiency of their stabilization. 0.15% SDS and 2% AgA have the highest optical density in the wavelength range from 400 to 900 nm, which corresponds with the literature [42, 43]. Ag NPs, stabilized by various polymeric compounds, however, have the absorption peak in the 360–430 nm region, typical for silver [1, 37]. As it follows from the Fig. 2 b,c, the lowest absorption peak wavelength is 372 nm for Ag + PVA. It corresponds to the smallest diameter of particles [41], while Ag + Ole Na, CMC and SDS possess peaks at 402, 409 and 410 wavelength respectively, where diameter of nanoparticles is a shade more. The spectrum for Ag + AgA doesn't have any peaks and is the same as for AgA absorption (Fig. 2 a). The absence of any peaks in the 360–430 nm region can be explained by the weak stabilization properties of agarose, intensive nanoparticle clustering and, as a result, faint and red-shifted absorption peak, that is much less than the agarose absorption at these wavelength and thus invisible on the spectrum.

The possible reason of such inefficient stabilization by agarose – its high molecular weight. It can provide clustering of silver nanoparticles rather than their isolation from each other, and creates high ζ - potential of such aggregates. In comparison to agarose, other stabilizers have the molecular weight at least 2 times lower – which allow them to stabilize silver nanoparticles more efficiently.

**Fig. 3:**
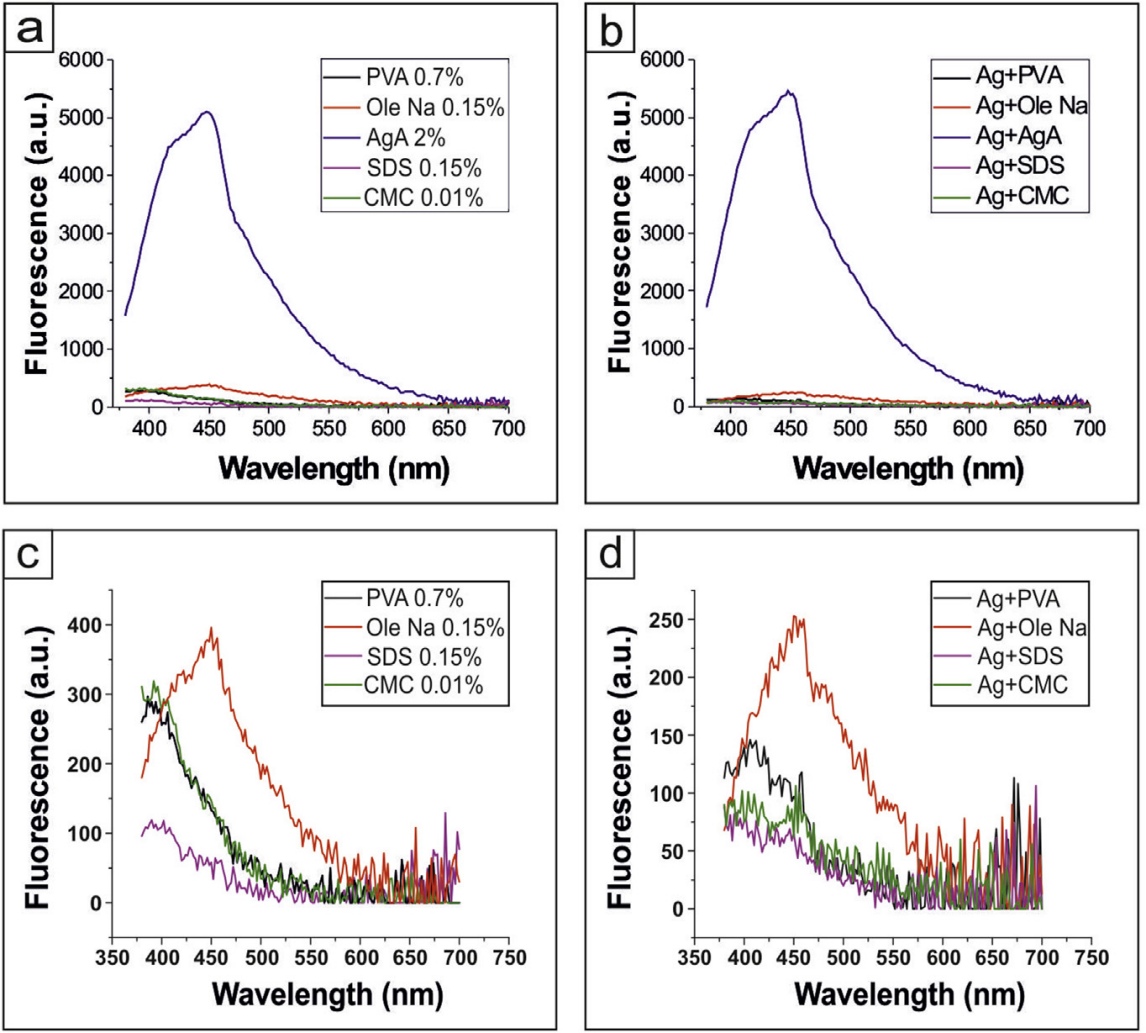
Fluorescence spectra of different polymer compounds (a,c) and silver nanoparticles, stabilized by them (b,d).

Fluorescence spectra of stabilizers and stabilized nanoparticles were carried out in the range from 380 to 700 nm with the excitation at 350 nm. We found that fluorescence spectra of 2% AgA (Fig. 3a), as well as Ag NPs, stabilized by 2% AgA (Fig. 3b), are similar with the emission peak at 450 nm. Other investigated stabilizers had no specific emission peaks (Fig. 3c,d).

**Fig. 4:**
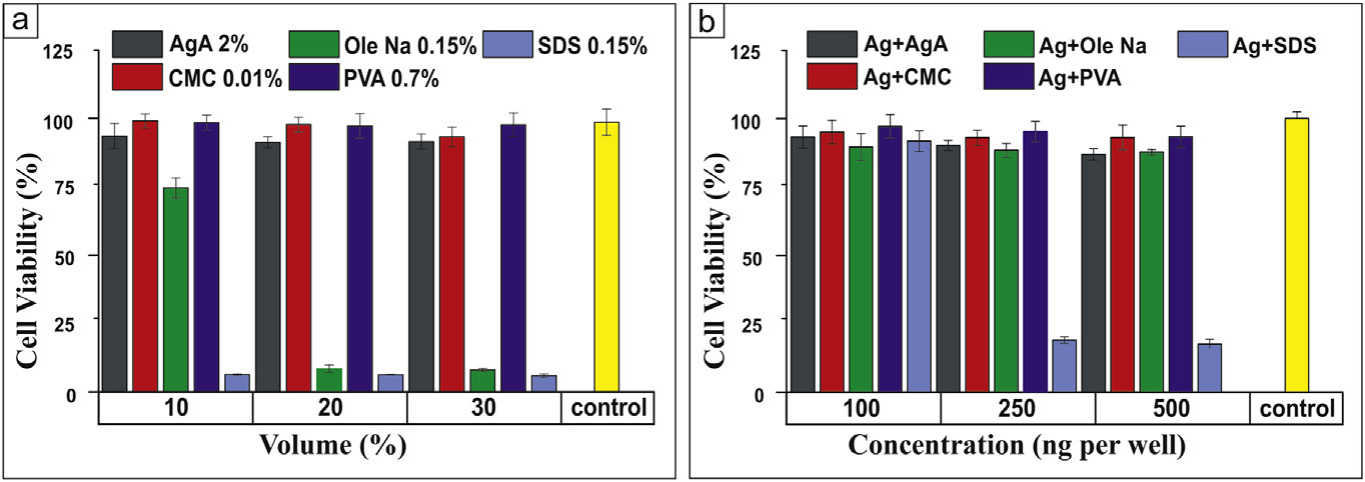
Cytotoxic effect of different polymer compounds (a) and silver nanoparticles, stabilized by them (b).

In order to examine the safety of the stabilizers above, we evaluated the cytotoxicity of stabilizers and stabilized Ag NPs on the NHDF cell culture. First, stabilizers were added to the cells in the amount of 10, 20 and 30% of the nutrient medium volume. We found that 0.15% SDS possesses the greatest cytotoxic effect. In all three concentrations, it led to 90% decrease in cell viability. The 0.15% solution of Ole Na had the cytotoxic effect as well. But this solution led to 90% decrease in cell viability at the amount of 20 and 30%. The 10% concentration of Ole Na resulted in a slight inhibition of metabolic activity. Other investigated stabilizers did not reduce to a significant decrease in cell viability (Fig. 4 a).

Then we added the stabilized Ag NPs to NHDF cell culture to test their toxicity. 100, 250 and 500 ng of nanoparticles per well did not provide a cytotoxic effect at all except for nanoparticles, stabilized by 0.15% SDS. 250 and 500 ng per well concentration of Ag NPs, stabilized by 0.15% SDS, led to 80% decrease in cell viability. The 100 ng per well concentration did not reduce to significant inhibition of the cell's metabolic activity (Fig. 4 b).

## 4 Conclusions

Ag NPs are widely used in many fields of industry due to their antibacterial properties. However, some nanoparticles are able to aggregate during their production and usage, which can lead to a decrease in their biological activity. In addition, Ag NPs can provide the cytotoxic effect on eukaryotic cells. With this regard, there is a need to search for new stabilizers for silver nanoparticles, which could prevent their aggregation and decrease their cytotoxic effect. In the course of this study, we estimated some physical properties and cytotoxicity of several polymeric compounds that can be used further as stabilizers of Ag NPs. Based on the results, 0.15% solution of PVA and 0.01% solution of CMC showed a good potential as stabilizers of particles. These polymeric compounds did not lead to aggregation of Ag NPs and did not provide the cytotoxic effect on the NHDF cell culture. Therefore, they are commonly used in many fields of biomedical application: ophthalmology, drug delivery [44, 45], as well as in food industry and cosmetics [46]. 2% solution of AgA did not provide a cytotoxic effect as well due to its natural origin [47], but it caused to particle aggregation. 0.15% solutions of Ole Na and SDS, to a variable degree, prevented them from aggregation, but exhibited the cytotoxic effect on fibroblasts. It occurs due to the threshold of concentration, depending on the cell type, upon which these solutions lead to inhibition of cells activity [48, 49, 50]. Thus, 0.15% solution of PVA and a 0.01% solution of CMC can be recommended for further usage as Ag NPs stabilizers due to the high effectiveness of stabilization and safe application. Based on the results above, further in vitro and in vivo experiments can be carried out without any significant side effects, caused by stabilizer.

## Declarations

### Author contribution statement

Roman Verkhovskii: Conceived and designed the experiments; Performed the experiments; Analyzed and interpreted the data; Wrote the paper.

Anastasiia Kozlova: Performed the experiments; Analyzed and interpreted the data; Wrote the paper.

Vsevolod Atkin, Roman Kamyshinsky: Performed the experiments; Contributed reagents, materials, analysis tools or data.

Tatyana Shulgina: Contributed reagents, materials, analysis tools or data.

Olga Nechaeva: Conceived and designed the experiments; Contributed reagents, materials, analysis tools or data; Wrote the paper.

### Funding statement

This research did not receive any specific grant from funding agencies in the public, commercial, or not-for-profit sectors.

### Competing interest statement

The authors declare no conflict of interest.

### Additional information

No additional information is available for this paper.
